# Language Comprehension in Background Noise – Effects of Noise Type and Task Modality

**DOI:** 10.5334/joc.478

**Published:** 2026-01-07

**Authors:** Michaela Socher, Isabella Ström, Josefine Andin, Åsa Elwér, Åsa Wengelin, Elisabeth Ingo

**Affiliations:** 1 Fraunhofer Institut of Building Physics, Nobelstr. 12, 70569 Stuttgart, Stuttgart, Germany; 2 Linköping University, 581 83 Linköping, Linköping, Sweden; 3 University of Gothenburg, Renströmsgatan 6, 405 30 Gothenburg, Gothenburg, Sweden

**Keywords:** Attention, Learning, Reading, Sentence, processing, Speech perception

## Abstract

In school, children often have to read or listen to spoken language while background noise is present. However, previous studies show a negative influence of background noise on reading and listening comprehension, especially in children. These background noise effects are not solely due to masking effects. Two accounts used to explain background noise effects are the interference-by-process account and the renewed view of age-related distraction. The interference-by-process assumes that the overlap of the processes needed for the background noise and the focal task are of importance, while the renewed view of age-related distraction argues that the modality of the focal task needs to be taken into consideration. In this study a total of 125 fifth grade students completed both listening and reading comprehension tasks under three listening conditions: silence, semantic noise and non-semantic noise. We found significantly lower performance for the semantic background noise condition compared to the silent condition. There was no significant interaction between task modality and background noise. These results are broadly consistent with the interference-by-process account but provide no support for the specific modality-based prediction derived from the renewed view of age-related distraction.

## Introduction

The learning environment of school-aged children is characterized by a considerable amount of background noise: children or the teacher talking or walking around, noise from other classrooms, traffic noise from outside, etc. In this noisy environment, children are expected to learn, despite research showing a negative influence of both chronic and acute noise exposure on academic performance (Shield & Dockrell 2003). Learning in a school context is highly dependent on comprehending language (both spoken and written). Studies indicate a negative effect of background noise on both listening (Klatte et al. 2013) and reading comprehension (Vasilev et al. 2018), but our knowledge about the underlying mechanisms behind this is limited. Language comprehension in school-aged children has mainly been studied in the silent condition (Cain et al. 2004; Kim 2015). This study aims to investigate two theoretical frameworks which explain the influence of background noise on language comprehension: *the interference-by-process account* (Marsh et al. 2009) and *the renewed view of age-related distraction* (Guerreiro et al. 2010).

Being able to read and comprehend is often described as prerequisites for educational success and has been investigated in many studies. Theories of comprehension, which stem from the area of reading comprehension, suggest that language (be it written or spoken) is processed in the form of statements, loosely defined as a clause (Kintsch & van Dijk 1978; Kintsch & Mangalath 2011). Understanding entails building a network of relationships between the statements and knowledge of the reader, i.e., making inferences. Language comprehension is often described as a domain-general skill, which means that the process of comprehension is the same independently of the modality used to present the material (Gernsbacher et al. 1990). The few studies that have used parallel tests and formats of reading and listening comprehension have found that the skills are similar but not entirely overlapping (Diakidoy et al. 2005; Vellutino et al. 2007). According to the simple view of reading, reading comprehension is the product of decoding and listening comprehension (Gough & Tunmer 1986). Reading comprehension is mostly determined by decoding level in inexperienced readers, but as children become more proficient decoders, reading comprehension, and listening comprehension are highly correlated (Catts et al. 2006).

Background noise reduces speech intelligibility and therefore makes listening comprehension more demanding. In accordance with this, studies have found reduced speech perception ability due to background noise in children (MacCutcheon et al. 2019). Studies even indicate that the signal-to-noise ratio (difference in sound level between target speech and background noise) needs to be higher for children than for adults for speech to be perceived as intelligible (Klatte et al. 2013). However, children show a reduced language comprehension ability in the presence of background noise if speech perception is controlled for (Klatte et al. 2010a; Valente et al. 2012) and even in conditions with perfect speech intelligibility (Klatte et al. 2007). Also, previous work by Dockrell and Shield (2006) and a meta-analysis by Vasilev et al. (2018) show that background noise has a negative effect on reading comprehension. Studies by Vasilev et al. (2019) and Meng et al. (2020) indicate that background noise has a negative effect on the reading process. Therefore, the effects of background noise on language comprehension cannot solely be explained by reduced speech perception ability due to masking.

According to the inference-by-process account (Marsh et al. 2009), a disturbance of background noise is expected if the background noise and the focal task compete for processing resources (Hughes 2014). If the background noise occupies similar processes as the focal task, this will lead to reduced processing capacity (Hughes 2014). For example, if a child is reading while someone is talking in the background, both the reading task and the background speech require language comprehension processes. The background speech would be automatically processed, introducing an additional source of semantic information and therefore a potential source of disruption to reading comprehension in comparison with performance in silence. This is in accordance with the results by Vasilev et al. (2018) and a recent study by Guerra et al. (2021), showing that intelligible background speech has the biggest interference effect on reading comprehension of all background noise types in both children (Vasilev et al. 2018; Guerra et al. 2021) and adults (Vasilev et al. 2018). Also, Klatte et al. (2010a) showed that listening comprehension of school-aged children is more disturbed by background speech than by other types of classroom noise. According to the inference-by-process account (Marsh et al. 2009), the increased disruptive potential of background speech on language comprehension in comparison to other types of background noise can be explained by a higher degree of shared processes. Background speech has semantic content which is processed automatically. Therefore, if children are engaged in a language comprehension task while listening to background speech, they need to process both the semantic content from the focal task and the semantic content from the background speech. This might explain why background speech has been found to be more detrimental than other noise types (Vasilev et al. 2018; Guerra et al. 2021; Klatte et al. 2010a).

Studies on developmental change show that background noise effects on listening comprehension change with age (Elliott 2002; Klatte et al. 2010b). Schwarz et al. (2015) argue that these results can be explained by changes in attention control. This is in accordance with results by Elliott et al. (2016), who showed that children compared to adults are more disrupted by background noise of any type when engaging in a visual serial recall task. Also, Joseph et al. (2018) found developmental differences between children and adults in terms of background noise effects on visual short-term memory for disruption caused by a deviant sound. In an updated version of the interference-by-process-account, the duplex-mechanism account (Hughes 2014), it is argued that both overlapping processes and attention capture can lead to disruptive effects of background noise on a focal task. However, a study by Röer et al. (2018) did not find age related changes in terms of the auditory deviant effect on visual short-term memory and a meta-analysis by Vasilev et al. (2018) did not find any differences in terms of the effect size of background noise effects on reading comprehension of children and adults. Röer et al. (2018) argue that the absence of age-related changes of background noise effects on a visual task might be explained by lower inhibition demands due to the focal task (visual) being in a different modality than the background noise (auditory). This argument is following the renewed view of age-related distraction (Guerreiro et al. 2010).

The renewed view of age-related distraction (Guerreiro et al. 2010) was originally developed to explain differences in divided attention ability between younger and older adults. It is argued that when background noise is present during a task, the participants have to inhibit the irrelevant information while focusing on (enhancing) the relevant information. This account does not assume that overlapping processes needed for the background noise and the focal task play a role. Therefore, this account does not make any clear predictions concerning which type of background noise would be most detrimental for specific focal tasks. Age-related changes in the influence of background noise in accordance with this view would be explained by differences in inhibitory control. This parallels the explanation offered by the duplex-mechanism account (Hughes 2014). However, the renewed view of age-related distraction (Guerreiro et al. 2010) makes a distinction between cross-modality of the focal task and the distractor and uni-modality of focal task and distractor. Guerreiro et al. (2010) argue that cross-modality of focal task and distractor facilitate inhibition ability. According to them, both the enhancement of the target modality as well as the inhibition of the distractor modality work together to minimize the distractor effects. This is in accordance with neuroimaging studies which have found increased activity in the part of the sensory cortex processing the target modality (Weissman et al. 2004) and suppression of activity in the parts that process the distractor modality (Johnson & Zatorre 2005).

In this study, we aim to investigate the influence of background noise type, as well as focal task modality on the effects of background noise on language comprehension in children. To our knowledge, no previous study has investigated the combined influence of noise type and task modality. Therefore, it is not known how background noise type and task modality interact and if these patterns can be explained by the interference-by-process account, the renewed-view of age-related distraction or a combination of the two. A greater influence of background noise with semantic content compared to background noise without semantic content would be expected according to the interference-by-process account. In this view, however, the modality of the focal task should not play a role in the disruptive effect of any background noise type. In comparison, according to the renewed view of age-related distraction it would be assumed that cross-modality of focal task and distractor leads to a reduction of background noise effects independent of background noise type (with or without semantic content). If, in addition, semantic noise is more detrimental to auditory compared to visual tasks, it would suggest that a combination of the two accounts might explain the influence of background noise on language comprehension. The results of this study can be used to expand theory development in the research area of background noise effects on children and inform educational strategies related to language comprehension. If the results show that reading and listening comprehension are equally negatively affected by low level background noise, recommendations for classroom organization as well as for school buildings need to be developed accordingly. For example, sound insulation between classrooms might need to be improved as a Swedish study shows that, in many schools, children hear teachers talking from adjoining classrooms (Brännström et al. 2017).

## Method

### Participants and Power Calculation

The participants in this study were typically developing children, without hearing impairment, dyslexia, or other neuropsychiatric diagnoses according to caregiver reports. All children had Swedish as one of their first languages and had spent their entire schooling in a Swedish school. They were attending the 5th grade of the Swedish basic education, which corresponds to being 11–12 years old. The data was used in a repeated-measurement ANOVA with two factors, modality, and noise. Modality had two levels (read, listen) and noise had three levels (silence, semantic, non-semantic).

To calculate the required number of participants, we simulated the intended design using the R (R Core Team 2021) package Superpower (Lakens & Caldwell 2021). For reading, we assumed small effects of non-semantic background noise and a slightly larger effect of semantic background noise in accordance with previous research (Vasilev et al. 2018). We assumed the same pattern for listening (Marsh et al. 2008), however, we assumed a generally larger effect of background noise on listening than on reading comprehension based on previous research Murphy et al. (2006). We assumed the correlation between the within subject levels to be high (r = 0.7) as research has shown that reading and listening comprehension are highly similar skills (Diakidoy et al. 2005; Vellutino et al. 2007). The effect sizes (partial eta-squared) used for the calculation were 0.12 for the main effect of modality, 0.19 for the main effect of noise, and 0.06 for the interaction between noise and modality. For the pairwise comparisons for the modality ‘reading’ the effect sizes (cohen’s d) were 0.24 for silence vs. semantic noise, 0.12 for silence vs. non-semantic noise, and 0.12 for semantic noise vs. non-semantic noise. For the pairwise comparisons for the modality ‘listening’ the effect sizes (cohen’s d) were 0.72 for silence vs. semantic noise, 0.33 for silence vs. non-semantic noise, and 0.40 for semantic noise vs. non-semantic noise.

**Figure:**
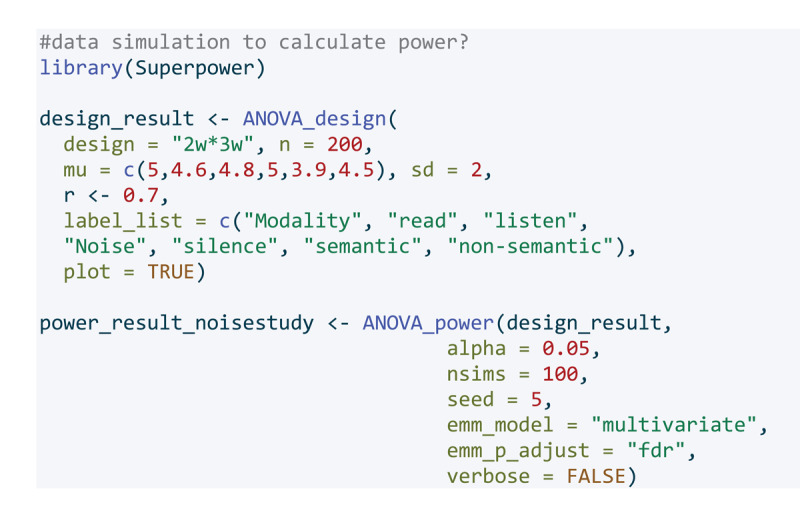


**Figure 1:**
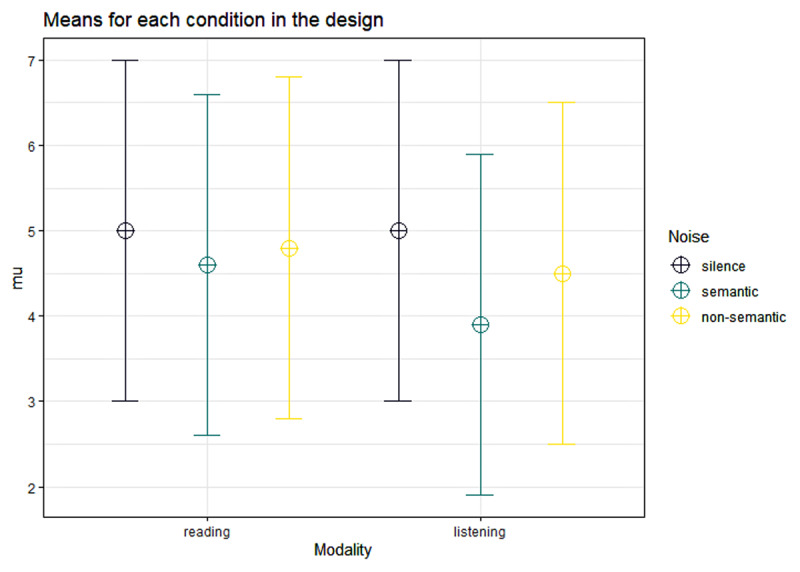
Simulated data for power calculation of the 2 × 3 ANOVA.

The data simulation is shown in Figure 1. We ran the simulation 100 times and subsequently calculated how many participants we would need to have an estimated power of 90% for detecting an effect of the expected size.

**Figure:**
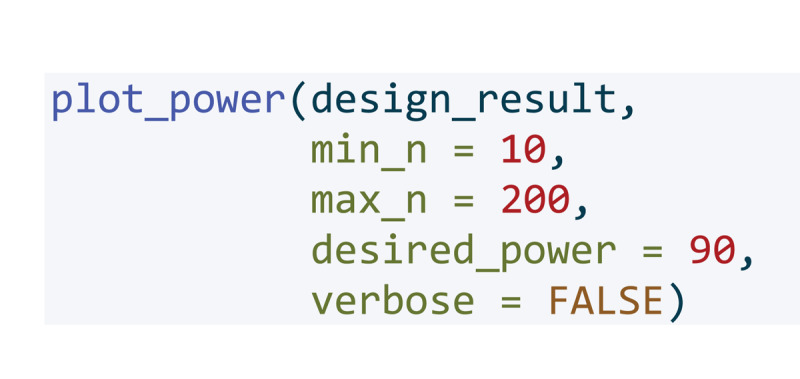


**Figure 2:**
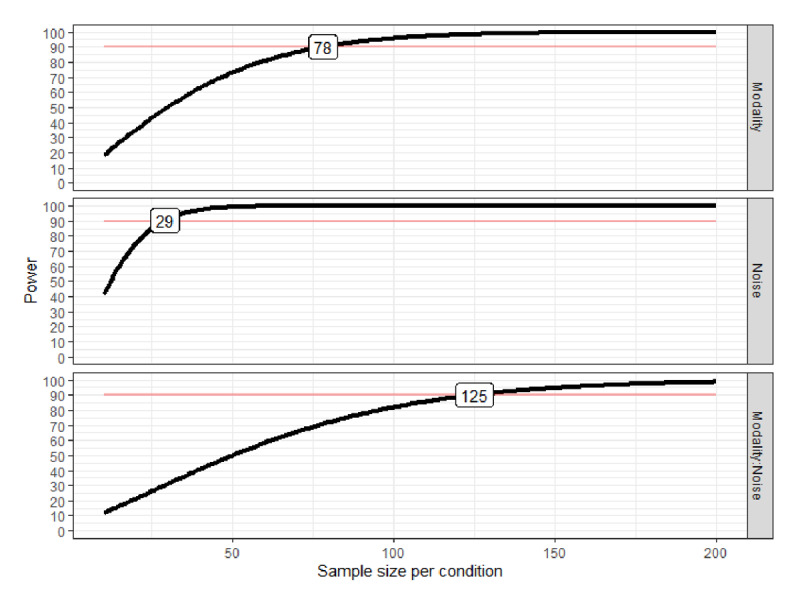
The red line indicates the target power and the black line indicates the expected power based on sample size. The numbers displayed on the respective panel show the sample size needed to reach 90% power for that effect.

As shown in Figure 2, 29 participants would be needed for 90% power to detect a main effect of noise of the expected size, 78 participants would be needed for 90% power to detect a main effect of modality of the expected size, and 125 participants would be needed for 90% power to detect an interaction effect of noise and modality of the expected size.

125 fifth graders were included in the final dataset (58 were boys, 65 were girls, and 2 chose not to disclose their sex). Their mean age at the first measurement point was 11 years and 7 months (SD = 4 months; range = 10 years 10 months – 12 years 3 months). Age data were not available for three participants who did not provide this information. As planned, children with a score of 0 in the silent condition were excluded from the dataset. This led to a final dataset of 122 children. However, as can be seen in Figure 2, this small deviation from the initially planned dataset size is unlikely to have a meaningful effect on statistical power.

### Ethical considerations

The study was approved by the Swedish ethical review authority (Dnr 2022-07060-1). All collected data was pseudonymised by assigning codes to each participant. Caregivers and participants gave informed consent and both participants and caregivers were informed that they could cancel their participation in the study at any time. Individual test scores of children were not shared with teachers or caregivers. All investigations were conducted following the tenets of the Helsinki Declaration.

### Measurements

All participants underwent language comprehension tests (listening and reading) under three conditions: silence, background noise without semantic content, and background noise with semantic content. Caregivers were asked to fill out a form with demographic questions including questions concerning hearing impairment, dyslexia, and neuropsychiatric diagnoses.

#### Language comprehension test

Six texts from the Swedish translation of the test I-Rest (Trauzettel-Klosinski & Dietz 2012) were used. The texts are simple expository texts, each of which presents facts about nature and animals. All texts consist of 829 characters including line breaks, 684 letters, 146 words, 9 sentences, and 16 lines. All texts have a readability index (Swedish LIX score) of 35. Text variability of the texts according to Type/Token Ratio (TTR), the Word Variation Index (WVI), and the Word Variation Ratio (WVR) are also comparable, and the same is the case for part of speech distribution. The same texts were used both for the reading and the listening comprehension test counterbalanced across task conditions. For the listening comprehension tests, the texts were recorded, spoken by a first language speaker of Swedish. In the original version of I-Rest, five multiple choice questions about the content are asked. These questions are all literal and for the current study two inferential questions were added per text. The principles used when formulating the inference questions were a) the question should not overlap with the literal questions b) the answer to the questions should not be explicitly stated in the text. The alternatives followed the same format as the explicit questions, 5 alternatives with “none of the above” included as the last alternative. The distractors were formulated to be likely given the text content by using formulations from the text which were not an answer to the question or likely given prior knowledge of animals and nature. Of course, none of the distractors were true. The test material was piloted before the main data collection took place to ensure equal task difficulty across reading and listening task and to ensure that the new questions and alternatives are valid.

For the listening comprehension test, the children listened to an audio recording from the I-Rest text material. They subsequently received an answering sheet with questions about the text (5 literal and 2 inferential questions). They read the questions and answered them in written form. In the noise conditions, no noise was present while the children read and answered the questions. The listening comprehension test was presented at a “normal speech level”, which was determined during piloting to be 56.5 dBA.

For the reading comprehension test, the children read a text from the I-Rest text material. They subsequently received an answering sheet with questions about the text (5 literal and 2 inferential questions). They read the questions and answered them in written form. In the noise conditions, no noise was present while the children read and answered the questions.

#### Exploratory analysis: Reading time measurement

As an exploratory measure, we also recorded participants’ reading time for the texts in I-Rest. Timing started when the participant began reading and stopped when the participant marked an “X” on the response sheet. The measurement was taken manually using a stopwatch application.

#### Background noise

Two kinds of background noises were used: one in which the semantic information was comprehensible and one in which it was not comprehensible. The background noises were created in a way that made their auditory properties as similar as possible. For the listening comprehension task, the target speech and background noise were mixed with a speech to noise ratio (SNR) of +23 dB S/N(A). Bradley and Sato (2008) have shown that this SNR leads to a ~99.5% speech intelligibility score for children around age 11. The children did listen to the noise, as well as to the noise + speech (in the listening condition) via headphones. To make the conditions as comparable as possible, the children also wore headphones in the silent condition.

##### Background noise with semantic content

For the background noise with semantic content, recorded snippets from a Swedish children radio program were used. Approval from the Swedish Radio for using the snippets in a research project has been obtained. The snippets included several children as well as an adult talking. Only snippets with a clearly intelligible dialog, without speakers masking each other, were used as a background noise for the condition ‘background noise with semantic content’. The snippets did not include any music.

##### Background noise without comprehensible semantic content

In order to make the background noise conditions as comparable as possible, the background noise used for the background noise with semantic content was used to create unintelligible multi-talker babble. This ensured a high similarity of the acoustic properties of the two background noises (Silbert et al. 2014). Also, multi-talker babble has a high ecological validity (Silbert et al. 2014). It is most likely a sound that the tested children have been exposed to in everyday live. Therefore, the advantage of using this kind of background noise, instead of inverted speech or speech-shaped noise, was the reduction of the unfamiliarity of the background noise. The noise was created by mixing snippets. Creating and evaluating the noise condition was part of piloting the material. We started with mixing 8 snippets, adding more until no speech was comprehensible but the noise was still recognized as containing speech, resulting in a final mix of 12 snippets.

### Procedure

Participants were recruited through contact with local schools. Teachers and other stakeholders were mediating information about the study and participation to caregivers. A questionnaire was filled out by the caregivers at home and returned along with the signed consent form for study participation. The children were tested individually in a quiet room at their own school. Testing took around two hours, as additional measures not reported in the current study were gathered. The test session was split into two parts. To account for order effects, the listening and reading comprehension test was block randomized. Half of the children did the reading comprehension test first and the other half did the listening comprehension test first. To account for order effects of the background noise conditions, the background noise conditions were randomized using a latin square design.

### Statistical Analysis

For the data analysis, R (R Core Team 2021) with the packages included in the tidyverse (Wickham et al. 2019), as well as the packages r-statix (Kassambara 2021), ggpubr (Kassambara 2020), xlsx (Dragulescu and Arendt 2020), gtsummary (Sjöberg et al. 2021), knitr (Xie 2014), kableExtra (Zhu 2021), BayesFactor (Morey & Rouder, 2022), and papaja (Aust & Barth 2022) were used.

A repeated-measures ANOVA with two within factors (modality: read, listen; Noise: silence, semantic, non-semantic) was used to analyse the data. To answer the research question, if noise with semantic content has a more detrimental effect on language comprehension than noise without semantic content, the main effect of noise was analysed. As there was a significant main effect of noise, a post hoc analysis using paired t-tests was used to evaluate which noise condition has the strongest effect. To evaluate this the following conditions were compared: silence vs semantic, silence vs non-semantic, semantic vs non-semantic. The false discovery rate approach (FDR) was used to correct for multiple comparisons. To answer the research question if the modality of the focal task influences the noise effects, the interaction effect of the factors noise and modality was analysed. As there was no significant interaction, simple main effects were not investigated. In case of a significant effect paired t-tests would have been used to evaluate if there is a significant difference of the influence of noise with semantic or without semantic content on reading in comparison to listening comprehension. Again, the false discovery rate approach would have been used to correct for multiple comparisons. In order to aid the interpretation of ambiguous findings, Bayes factors (BF10) were calculated for the noise and the modality effects. The detailed analysis script as well as the raw data, the meta data and the lab log can be found at https://osf.io/3srgd.

An exploratory analysis investigating differences in reading time between the different noise conditions was added after data collection was finished. A repeated-measurement ANOVA with one factor (Noise: silence, semantic, non-semantic) was used to analyse the data. Since there was a significant main effect of noise, we conducted a post hoc analysis using paired t-tests to evaluate which noise condition had the strongest effect. To evaluate this, the following conditions, were compared: silence vs semantic, silence vs non-semantic, semantic vs non-semantic. The false discovery rate approach (FDR) was used to correct for multiple comparisons.

## Results

Before conducting the main analyses, the data were screened for normality. Visual inspection of histograms and skewness statistics indicated no substantial deviations from normality, and therefore, no data transformations were deemed necessary (see analysis script). Three participants were excluded from the final analysis due to scoring 0 on the language comprehension task in the silent condition. These cases were considered outliers, as performance at floor level in the silent condition suggested disengagement or misunderstanding of the task.

**Figure 3:**
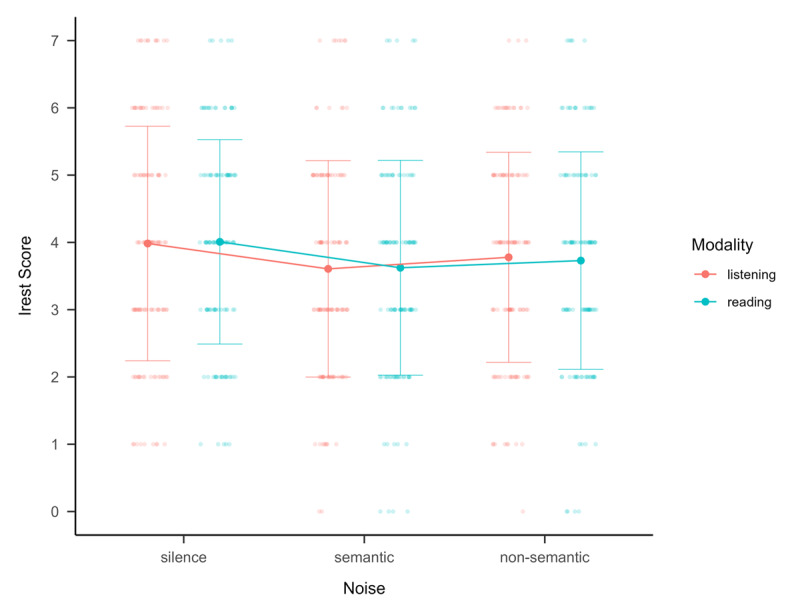
Performance for silence, semantic and non-semantic noise across the listening and reading conditions. The means and standard deviation for each condition are displayed together with the individual data. The data is grouped by modality.

Analysis is based on scores representing how well the participants answer questions on language comprehension tests, while exposed to different noise types (see Figure 3).

The repeated-measures ANOVA revealed a significant main effect on noise, *F* (2, 242) = 4.053, *p* = .019, η_G_^2^ = .01. A post hoc test, using FDR to correct for multiple comparisons, revealed significantly lower performance in the semantic noise (*M* = 3.61, *SD* = 1.60) compared to the silent condition (*M* = 4.00, *SD* = 1.63), *t*(121) = 2.8813, *p* = .013, *d* = .18 [.06, .31]. The difference between non-semantic noise (*M* = 3.75, *SD* = 1.59) and silence was not significant, *t*(121) = 1.9468, *p* = .079, *d* = .12 [–.01, .25], neither was the difference between semantic and non-semantic noise, *t*(121) = –1.0659, *p* = .288, *d* = –.0682 [–.19, .06].

There was no significant main effect of modality, *F* (1, 121) = .0007, *p* = .978, η_G_^2^ < .001, and no significant interaction of noise and modality, *F* (2, 242) = .0540, *p* = .947, η_G_^2^ < .001.

The comparison between silence and semantic noise resulted in a Bayes Factor (BF10) of 4.05 ± .01%. This means that the probability of a difference between the two conditions is around 4 times greater than the probability of no difference between the conditions. For the comparison between silence and non-semantic noise, the Bayes Factor (BF10) was .459 ± .05%, for the comparison between the semantic and non-semantic noise the Bayes Factor (BF10) was .125 ± .16%. In sum, only the comparison between silence and semantic noise yielded a Bayes factor indicating a higher probability of a difference between conditions than of no difference. The other comparisons did not show evidence of a difference.

For the comparisons between listening and reading, all Bayes Factors (BF10) were < .11. In other words, there was no indication of any difference in performance due to the different modalities (reading vs. listening).

### Exploratory analyses

Since the semantic noise condition was shown to be more difficult than the other conditions, we performed exploratory analyses to investigate whether the poorer performance was associated with slower reading speed, which could indicate pauses and re-reading of passages due to interference with the semantic from the noise. Visual inspection of histograms indicated a slight skew of the data. However, the skew was not deemed severe enough to indicate any data transformation.

**Figure 4:**
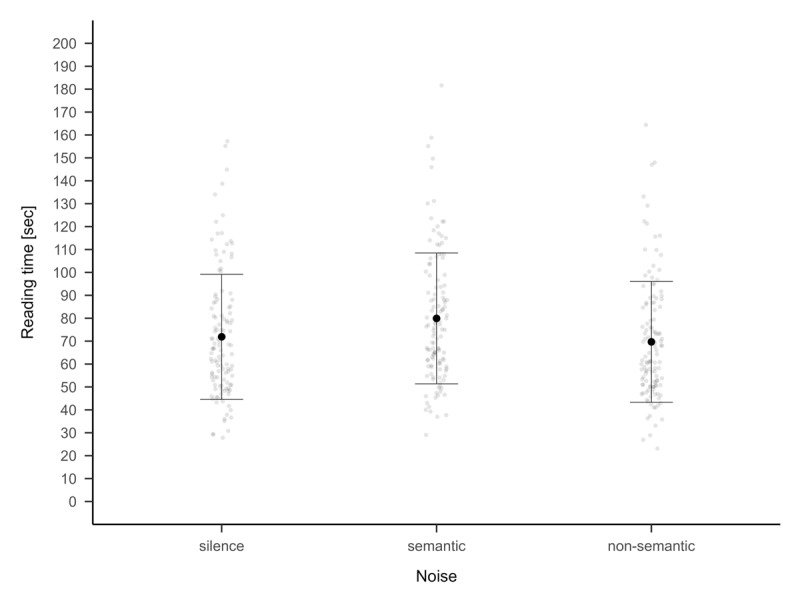
Reading time during silence, semantic and non-semantic noise for the reading task. The means and standard deviation for each condition are displayed together with the individual data.

A repeated-measures ANOVA with noise (silence, semantic, non-semantic) as a within-subjects factor revealed a significant main effect of noise on reading speed, *F*(2, 242) = 23.831, *p* < .001, η_G_^2^ = .025 (see Figure 4). Post hoc comparisons with the false discovery rate adjustment showed that reading speed was significantly slower in the semantic noise condition (*M* = 79.93, *SD* = 28.56) compared to both the silent condition (*M* = 71.89, *SD* = 27.30), *t*(121) = 5.3266, *p* < .001, *d* = 0.48 [.28, .74], and the non-semantic noise condition (*M* = 69.69, *SD* = 26.37), *t*(121) = 6.7180, *p* < .001, *d* = 0.61 [.41, .86]. The difference between silence and non-semantic noise was not significant, *t*(121) = 1.3386, *p* = .183, *d* = 0.12 [–.05, .31].

### Discussion

In this study, we investigated how different types of background noise influence language comprehension and whether this influence differs according to task modality (reading vs. listening). The results showed a significant decrease in performance due to semantic background noise both for listening and reading comprehension. Additionally, a Bayes factor analysis indicated a four times higher probability of a performance difference between comprehending language comprehension in silence and language comprehension with semantic background noise. Non-semantic background noise did not lead to a significant decrease in performance. Further, no interaction with task modality was found. The effect of semantic background noise on reading and listening comprehension was comparable. In sum, these findings are broadly consistent with the interference-by-process account (Marsh et al. 2009), suggesting that performance declines when the cognitive *processes* required by the focal task and the background noise overlap. However, the difference between the performance during semantic noise and the performance during non-semantic noise was non-significant. Therefore, noise with semantic content does not necessarily seem more disruptive than other noise types. As the *modality* of the focal task did not influence the magnitude of the effect of background noise on performance, our data do not support the modality-based prediction derived from the renewed view of age-related distraction for fifth-grade children (Guerreiro et al. 2010). However, this does not preclude the existence of modality-based effects in other age groups. In sum, our findings demonstrate that semantic background noise impairs language comprehension in children regardless of whether the task involves reading or listening, thereby providing support favoring the interference-by-process account.

The results of this study are in line with previous studies showing that semantic background noise has a larger effect compared to non-semantic background noise on listening comprehension, particularly in children (Klatte et al. 2010a), and on reading comprehension in both children and adults (Vasilev et al. 2018). Results by Hyönä and Ekholm (2016) suggest that if background speech can be semantically processed, it leads to greater disruption in adults (in their study: native language vs. foreign language). They argue that this is due to two sources competing for semantic processing resources. This mimics the explanation for background noise effects offered by the interference-by-process account. In this account, overlapping processes of the focal task and the background noise are postulated as a reason for background noise effects. Although their study focused on adults, it is reasonable to assume that similar mechanisms could also apply to children. Marsh et al. (2024) suggested that, in young adults, interference due to background noise may also result from temporal variation of the background noise or the need for sequential attention, even when the distractors are non-verbal. This would suggest that non-semantic background noise also shares processing resources with reading and listening comprehension. Therefore, a disruption of performance would be expected. However, we did not find support for this in the present study.

In contrast to the current study, previous studies have found negative effects of non-semantic background noise on listening comprehension in adults (Marsh et al. 2015) and reading comprehension in both children and adults (Vasilev et al. 2018). However, these effects are usually small (Vasilev et al. 2018), and in the case of listening comprehension, the lower SNRs used in studies can lead to an auditory masking effect. Such an effect would create difficulties in listening comprehension on the perception level (Klatte et al. 2010a; Carlie et al. 2024; MacCutcheon et al. 2019). In the current study, the background noise was presented with a SNR of +23 dB S/N(A). According to Bradley and Sato (2008) this should lead to a ~99.5% speech intelligibility score for children around age 11. Therefore, all effects of the background noise in this study can be attributed to interference at the cognitive rather than the perceptual level. This has likely reduced the magnitude of the effect in comparison to previous studies. Also, the non-semantic noise in the current study was mostly continuous and lacked temporal variation, such as pauses and variation in sound level. This might have reduced the automatic sequential processing and thereby the effect of the non-semantic background noise. More studies are needed to disentangle the different effects of temporal variation, semantic content, and noise level on listening and reading comprehension.

Semantic background noise had a significant effect on listening and reading comprehension in this study; however, the effect was small. The question arises whether the effect can still be considered relevant. We consider the effect educationally relevant despite its small size for the following reasons: Despite the short duration of the language comprehension tasks (30 seconds to 3 minutes), semantic background noise significantly impacted comprehension. Performance dropped on average by 0.38 points respectively, which is a decrease in performance of 9.5% on average compared to the silent condition. If this effect would be stable over time, an accumulated effect would be expected. However, further studies are needed to evaluate if the effects of low noise on language comprehension accumulate or if any habituation effect occurs over time. In a realistic classroom setting the background noise is usually louder (Lundquist, 2003; Fidêncio et al. 2024) compared to the current study. It is reasonable to expect that an increase in background noise level will lead to greater disturbance. Firstly, because the SNR will be reduced, and for listening comprehension disruption will already occur on the perception level. Secondly, because noise level has previously been found to be an important factor for the effect of background noise on reading. In a study of children aged 8–10 years, Guerra et al. (2021) found that louder background noise leads to greater disruption of reading speed compared to softer background noise. More studies are needed to evaluate the precise relation between increasing level of background noise and language comprehension. In the current study, background noise was only presented during reading and listening. The children answered the questions in silence. In a realistic classroom setting, this would not necessarily be the case. For adults, Sörqvist et al. (2012) have shown that background speech leads to a disturbance of the writing process. The same might be the case for children. More studies are needed to evaluate the combined effects of background noise on language comprehension and language production to evaluate which effects would be expected in a realistic classroom setting.

The effect of semantic background noise found in the current study was in accordance with predictions made by the interference-by-process account. However, as noted above, due to the lack of a robust semantic-non-semantic contrast, the support was only partial. Further, at which processing stage the interference occurs is currently unclear. Results by Hyönä and Ekholm (2016) indicate that participants reread complex sentences when exposed to semantic background noise. The authors argue that this might be a strategy to reactivate the working memory representation of the recently read sentence, which has been overwritten by the semantic background noise. In line with this interpretation, our results from the exploratory analysis show that semantic noise reduces children’s reading speed. These analyses were not preregistered and should therefore be regarded as exploratory and interpreted accordingly. Both reading speed and comprehension were negatively affected in the presence of semantic noise. This suggests children either spend additional time reading, decrease in their reading comprehension ability or possibly do both in the presence of semantic background noise. Wasiuk et al. (2021) found an effect of background noise on event model memory but not on surface form and propositional level for listening comprehension in young adults. Event model memory refers to the memory about what has been said, while the surface form refers to the exact words being used, and the propositional level refers to the idea units without the exact wording. The authors argue that more processing resources might be needed at lower levels of speech processing when background noise is present. This may lead to a lack of available resources for higher-order language processing. To our knowledge, there are currently no studies on children investigating at which level of language processing and memorizing background noise affects occur.

### Future research

In this study, we found that semantic background noise disrupted language comprehension in both reading and listening tasks among fifth-grade children. This suggests that meaningful background speech can interfere with how children process language, regardless of the input modality and even when language comprehension is not disturbed on a perceptual level (high SNR, children allowed to reread sentences). Also, the results show a disturbing effect of quiet semantic background noise for both reading and listening comprehension. Studies from different countries show that sound levels in unoccupied classrooms often exceed 30 dB(A) (Seitz & Fels, 2025; Sala & Rantala, 2016). Parts of this noise in unoccupied classrooms are caused by sounds from adjacent classrooms, which also entail speech. Research is needed to investigate at which levels background speech has a negative impact on language comprehension and how low the speech transmission index (STI) needs to be to ensure no negative effects. Such a study could be used to develop building acoustic recommendations for schools. In addition, studies are needed to investigate how building acoustic measures and/or schedule planning can reduce speech sounds from other classrooms.

So far, only a limited number of studies have investigated the influence of different types of background noise on listening and reading comprehension in children. The current study found evidence that is broadly consistent with the interference-by-process account as an explanation of background noise effects for children. A result also found in studies on other cognitive abilities such as short-term memory in children (Leist et al. 2022). However, several questions remain. It is currently unclear which long-term effects background noise would have on language comprehension in children. Also, the combined effects of noise during language comprehension and language production have not yet been examined and the specific processing level at which the disruption by background noise occurs is currently unclear. Most importantly, as can be seen in Figure 3, the performance of the different children shows a large variability in all noise conditions. The large variability in performance under the non-semantic noise condition suggests that individual differences may play a role for noise effects on language comprehension. This should be addressed in future research. Previous research in children has suggested that working memory capacity (Sullivan et al. 2015; McCreery et al. 2017, inhibition skills (Röer et al. 2018), and linguistic skills (Klatte et al. 2013; McCreery et al. 2017 influence the effect of background noise. More research is needed to investigate which children in different age groups are especially vulnerable to background noise effects and whether these effects are different in reading compared to listening tasks.

## Conclusion

The present study shows that semantic, but not non-semantic, background noise modestly but statistically significantly impairs language comprehension in fifth-grade children relative to silence. The noise effect did not differ by modality: reading and listening were affected to a similar extent. Given the favorable SNR used, the observed decrease in performance is best attributed to cognitive interference rather than reduced speech intelligibility and is broadly consistent with the interference-by-process account. In line with this interpretation, our exploratory analysis indicated that semantic background noise slowed down children’s reading pace, yet the slower pace did not translate into better language comprehension outcomes.

Although the effects of semantic noise on language comprehension are small, they are practically meaningful since they may accumulate over time and are likely to be amplified in typical, louder classroom environments. Further studies are needed to test this in more ecologically valid classroom-like conditions, to examine combined effects on comprehension and production, and to identify individual factors which may make children more susceptible.

## Data Accessibility Statement

All data analyzed during this study are available in the Open Science Framework (OSF) repository associated with the registered report: https://doi.org/10.17605/OSF.IO/J3BKR.
